# The F-words relating to symptomatic flexible flat feet: A scoping review

**DOI:** 10.1371/journal.pone.0320310

**Published:** 2025-05-07

**Authors:** Jovana Urukalo, Helen Banwell, Cylie Williams, Stewart C. Morrison, Saravana Kumar

**Affiliations:** 1 Allied Health and Human Performance, University of South Australia, Adelaide, Australia; 2 Monash University, School of Primary and Allied Health, 47-49 Moorooduc Hwy, Frankstown, Victoria, Australia; 3 Department of Population Health Science, Faculty of Life Sciences and Medicine, School of Life Course and Population Sciences, King’s College London, London, United Kingdom; Brunel University London, UNITED KINGDOM OF GREAT BRITAIN AND NORTHERN IRELAND

## Abstract

Flexible flat feet are one of the most common musculoskeletal concerns presenting to paediatric health services, despite this being an expected finding in children under 10 years and only requiring management when symptoms are associated. Understanding which symptoms are associated with symptomatic presentations of flexible flat foot in children will provide clarity in identifying those that require further assessment and/or intervention. A scoping review of the literature was conducted to gather all known symptoms related to symptomatic flexible flat foot in the child. Data was mapped using the ‘F-words’ framework, a child friendly, six-item tool based on the International Classification of Functioning, Disability and Health Framework 11 (ICF-11). This review identified 42 individual symptoms relative to symptomatic presentations of flexible flat foot, which were allocated into five of the six ‘F-words’ categories (*fitness, functioning, friends, family* and *future)*. Of these, pain was the most reported symptom, identified in 124 (of 133) included citations, followed by symptoms associated with reduced lower limb function (altered gait patterns, reduced balance and stability and increased tripping), fatigue and reduced participation. Other less frequently reported symptoms include callus formation, night pain and cramps. When present, these symptoms may occur independently or may co-exist at the same time. No symptoms were allocated to the *fun* category of the ‘F-words’. A multitude of symptoms are reportedly associated with symptomatic flexible flatfoot in the child, with no discernible pattern or coherence noted. Further research should examine development and progression of symptoms and seek to better understand causality of relationship between symptoms and foot posture.

## Introduction

Flexible flat feet (flat feet), also known as pes planus, are defined as feet with a lowered medial longitudinal arch (MLA) with or without hindfoot eversion [1,2]. Flat feet are reported in 48% to 77.9% of typically developing children [3,4], identified via a visible arch during non-weight bearing and no arch in weight bearing [5], and is an expected observation in children under 10 years [4,6,7]. Specifically, the flatness of a child’s feet generally decreases incrementally across the first decade of life [8,9], with most not requiring any clinical intervention [10,11], despite prevailing arguments in the literature [1,5]. Despite flat feet being typical in this population, they remain one of the most frequently presenting concerns to paediatric health services [8,12,13]. This is likely driven by concerns that having flat feet will negatively impact a child’s lower limb function, despite evidence suggesting only a small subset of children will develop symptoms associated with this foot posture [14]. However, when this concern is coupled with increasing availability of often incorrect or conflicting advice (e.g., via websites, social media and parents forums) [15], and a lack of clear guidelines on when we should and shouldn’t be concerned about flat feet, it is understandable that confusion still exists. Understanding which symptoms are reportedly associated with flat feet in children is the first step in unpacking how we can more clearly define ‘symptomatic’ presentations of flat foot and offers us the ability to design robust investigations to determine the strength of association, if any, between symptoms and foot posture. Ultimately, this can offer parents/carers and practitioners direction on when intervention may, or may not, be warranted.

Encouragingly, some headway has been made in identifying and defining ‘symptomatic’ versus ‘asymptomatic’ flat foot. Benedetti, Ceccarelli [16], conducted an observational cohort study of 53 children with flexible flat feet. Of the 53 children that were reported as symptomatic, clinical measures, functional tests and interviews identified that the children with symptomatic flexible flat feet reported early fatigue, and pain, particularly at the medial hindfoot and plantar aspect of the foot and observed a reduction in functional activities during prolonged standing or walking were most associated. Other studies have also reported symptoms relating to flat foot posture such as knee pain [17], generalised pain with activity [18], a reduction in lower limb function such as reduced balance and increased tripping [19,20]. Importantly, guidance has been developed for practitioners’ such as a validated proforma for identification for symptomatic (vs asymptomatic) with guidance on concern [14] and a protocol to facilitate when, why and how the prescribing of foot orthoses and other interventions may occur for children with flexible flat feet [21]. However, no research has captured an exhaustive list of symptoms reportedly associated with paediatric flat feet. With a clearer boundary and understanding around associated symptoms, an improvement of clinical decision-making surrounding treatment may be observed.

While there is currently no clear evidence to indicate that symptomatic flat feet in children progresses to symptomatic flat feet in adults, there are concerns about health consequences of flat feet in adults [22–25]. Understanding which symptoms reportedly impact children with symptomatic flexible flat foot may have an impact in later life and is the first step in unpacking when and why intervention may be beneficial to prevent future issues. Therefore, this scoping review aimed to synthesise known or reported symptoms associated with paediatric symptomatic flexible flat foot (symptomatic flexible flat foot) to provide recommendations for data collection in clinical practice and inform future research studies about which symptomology may be appropriate to capture.

The aim of this study was to conduct a scoping review of known or reported symptoms associated with paediatric symptomatic flexible flat foot (symptomatic flexible flat foot).

### Methods

This scoping review was underpinned by the methodology of Arksey and O’Malley [26] and the PRISMA-ScR reporting guidelines [27]. We used the World Health Organisation’s International Classification of Functioning, Disability and Health Framework 11 [28,29], or more specifically, against the ‘F-words’ version (f*unctioning, family, fitness, fun, friends,* and *future*), a framework commonly used to map function and disability in children [30], to map the commonly reported symptoms. The protocol was registered at Open Science Framework (OSF) in December 2021 (https://osf.io/pfbw7).

### Inclusion and exclusion criteria

Information related to children between the ages of 0–18 with symptoms reported to be related to symptomatic flexible flat feet that met the criteria were eligible for inclusion in this review (Table 1). For this review, any symptoms of symptomatic flexible flat feet as reported in the literature were eligible for inclusion. This was inclusive of quantitative, qualitative, and descriptive design, non-peer reviewed articles, blogs, and opinion pieces, with no limitation applied to study setting (e.g., clinic, research lab) or author qualifications.

**Table 1:** Inclusion and exclusion criteria.

Inclusion	Exclusion
Criteria relevant to study and publication information Symptomatic flexible flat feet Lower limb/back pain Fatigue Instability Ligament laxity Quality of life Reduced activity levels Clinical practice/community	Criteria relevant to study and publication information Asymptomatic flexible flat feet History of lower limb surgery* Infectious, genetic, or systemic conditions Neural or muscular abnormalities History of lower limb and/or foot fractures
Criteria relevant to participants Children 0–18 years	Criteria relevant to participants • Adults 19 + years

* Studies reviewing surgical management of flat feet were included if the above inclusion criteria were met and reported symptoms were captured prior to surgical intervention

### Search strategy

A search strategy was developed in conjunction with an Academic Librarian at University of South Australia. Commercially produced literature from electronic databases were searched from inception to November 2023: Medline (via Ovid), Emcare (via Ovid), Embase (via Ovid), The Cochrane Library, Scopus, UpToDate and SportsDiscus. The search included key words, Medical Subject Headings (MeSH) and free text terms for child/toddler/adolescent/youth and symptom/fatigue/pain/trip, flatfoot/flexible flatfoot/pes planus, truncated where relevant. Searches were limited to the English language and literature involving humans. The search terms employed for databases are included in S1 Appendix 1, 3–8. Grey literature was obtained via an internet search engine (Google and Google Scholar) using the following terms ‘symptomatic flat feet children’, ‘symptoms of pes planus in children’, ‘flat feet symptoms children’ and ‘children’s foot pain’, with the first ten pages reviewed. Pearling of all reference lists of included citations was also undertaken. The search was undertaken twice (23^rd^ November 2021 and 5^th^ November 2023) by one author (JU) and reviewed by two authors (HB/SK).

### Screening and selection of studies

All search results were exported to Endnote^TM^ (20.1) (Thomson Reuters, San Francisco, CA, USA), and duplicates removed. Screening of titles and abstracts, followed by full-text screening was completed in Covidence^TM^ [31]. Two reviewers (JU and SK/HB) independently assessed relevant studies against the eligibility criteria. Conflicts were resolved through discussion.

### Data extraction and synthesis

A purpose-built data extraction tool was developed for this review. It was piloted and modifications were made as required in Microsoft Excel (version 2103 © Microsoft Cooperation 2021). Data extracted included article information and demographics (author, year, country, study design, samples size), participant characteristics (age, sex/gender, ethnicity) and outcomes (e.g., signs and symptoms) as reported. All conflicts were resolved through discussion between the authorship team.

### Study mapping

‘F-words’ framework for Childhood Disability, a six-item tool, based on the World Health Organisation (WHO) International Classification of Functioning, Disability and Health Framework 11 (ICF-11) [28,29] was used to map the extracted data. The ICF-11 offers a unified language and a pragmatic approach to sharing standardized information as well as mapping data of a specific health condition and what impact that may have on function [32–34]. Appendix 9 provides an overview of data mapped against the ICF-11 domains and sub-domains of reported symptoms. After this, we used the F-words framework for Childhood Disability to further map the symptoms reported in the literature, considering the population of interest for this review (children). Rosenbaum and Gorter [30] suggested that applying the ‘F-words’ at the research, advocacy, and clinical levels allows service providers to effectively populate the ICF framework for children, thereby personalising interventions. Given the population of interest (i.e., children aged 0–18 years), this framework is particularly well-suited for paediatric topics. The ‘F-words’ offers six essential aspects of a child’s life. The ‘F-word’ *fitness* relates to the ICF-11’s body structure and function, referring to a child’s physical and mental wellbeing. The ‘F-word’ *functioning* relates to the ICF-11’s activity, referring what is important to a child and how things are done. The ‘F-word’ *family* relates to the ICF-11’s environmental factors, referring to a child’s essential environment. The ‘F-word’ *friends* relates to the ICF-11’s participation, referring to a child’s friendships established with those around them. The ‘F-word’ fun relates to the ICF-11’s personal factors, referring to activities that a child enjoys. The final ‘F-word’, future, refers to development, and the impact that the child’s current condition has on their future life [30]. The ‘F-words’ method of mapping is inspired by ICF-11, however, this framework focuses on all areas of a child’s development without placing increased importance on any one factor, allowing for the personalization of each individuals intervention [30]. Furthermore, this framework focuses on the strengths of each individual child, promotes a ‘can do’ approach to their everyday life and provides the ability to identify meaningful goals [35].

### Data synthesis

To ensure rigour and robustness of the mapping process, one reviewer (JU) independently mapped the data across four categories first. This was then followed by independent review from the remaining authorship group (SK, HB, SM, CW) and feedback provided. Conflicts were resolved through discussion.

## Results

### Study selection

**Fig 1:**
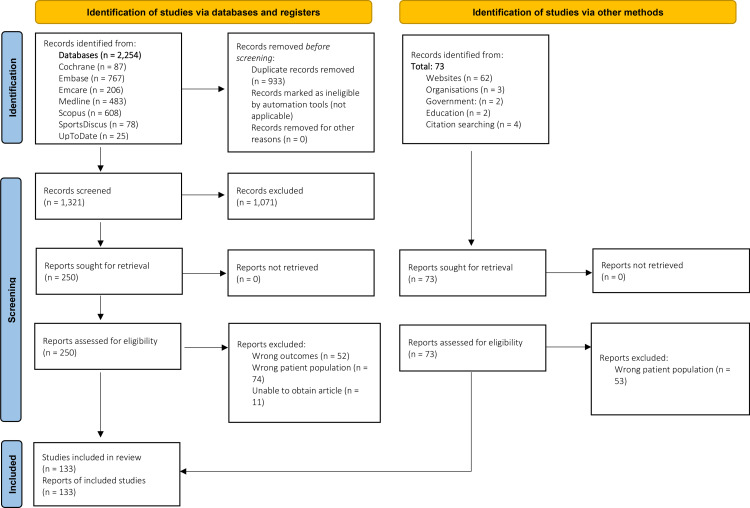
PRISMA Flowchart.

Searches returned 1321 unique citations, of which 133 were deemed eligible following full text screening (Fig 1). Of the 133 included citations, 113 were literature from electronic databases and the remaining 20 were grey literature from internet search engines. Citations were excluded for the following reasons: wrong outcomes (e.g., asymptomatic population) (n = 52), wrong patient population (n = 74), and unable to obtain article (n = 11) (webpage was no longer available or article unable to be retrieved).

### Study characteristics

The included citations originated from 29 different countries and were published as early as 1989, up to June 2023. A range of study designs were used (n = 11), with the most common being cohort studies (n = 36) (Appendix 2).

### Participant characteristics

A full review of participant characteristics is available in Appendix 2. Several citations reported in terms of number of feet as opposed to participants, where the number ranged from 5 participants to 2450 feet. Where age was reported, participants varied from 1–18 years of age. Two citations included age ranges from 4–20 years, respectively, [36,37] however, reported data for those aged 18 years or less separately. Many citations (n = 71) did not specify age range, instead identified the population as “paediatric”. Sex of participants were self-reported in 44 of the 133 included citations.

## Outcomes

**Fig 2:**
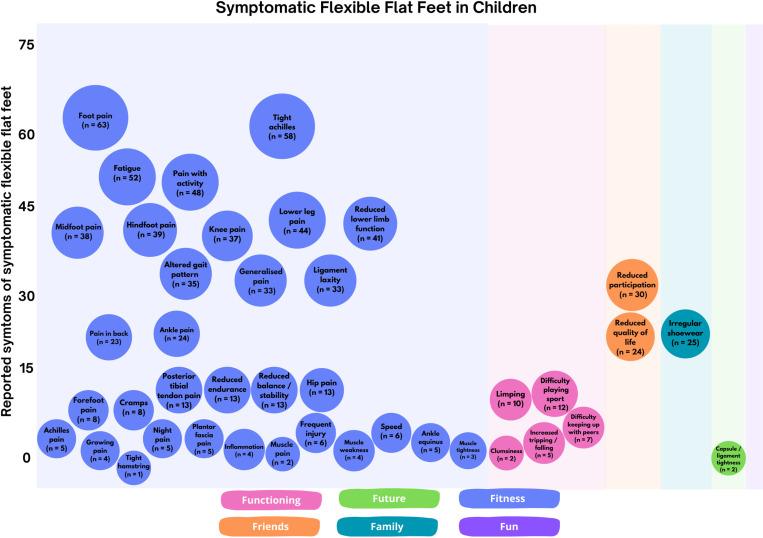
Symptoms mapped as per ‘F-words’.

Using the ‘F-words’ framework, of the 42 symptoms identified across 133 included citations, thirty-five (n = 35) could be mapped to *fitness* (body functions and structures), five (n = 5) to *functioning* (activity), two (n = 2) to *friends* (participation), one (n = 1) to *family* (environmental factors), one (n = 1) to *future* and none (n = 0) to *fun (*personal factors) (Fig 2 and Appendix 2). While most citations reported symptoms of symptomatic flexible flat foot, only a handful actually measured these.

### Fitness

Fitness was the most common domain reportedly impacted for children with symptomatic flexible flat feet, with symptoms associated to body function, and body structure related issues.

### Body function

#### Pain.

Pain was the most frequently reported symptom, reported in 124 (of 133) included citations. Pain was described via varied methods, however, could be broadly categorized into seven groups (Fig 2): *pain in lower limb, pain in joints, pain in multiple body parts, pain in back, generalised pain, sensory functions and pain* and *radiating pain in a segment or region.*

For the lower limb, foot pain was reported most frequently (n = 63) [1–4,9,21, 37–93], followed by lower leg pain (n = 44) [1,2,4,8,9,21,43,44,53,57,60,61,63,64,68,70,72,74–76,79,82,84–89,91,94–108]. One Delphi study seeking consensus of experts on symptoms associated with symptomatic flat foot [21] specifically noted medial tibial stress syndrome (MTSS) as an associated concern. Within the foot, the hindfoot was the most common area where symptoms presented (n = 39) [1,9,16,21,44,47,54,55,60–62,69,73,75,76,83,90,98,100,105–107,109–125], followed by the midfoot (n = 38) [13,21,37,44,47,54,55,57,58,60–62,64,73,76,83,88,97–99,103,106,107,110,112–115,117–121,124–128], forefoot (n = 8) [9,16,60,85,88,109,121,129] and then specifically within the plantar fascia (n = 5) [9,21,49,74,83]. Of the 39 citations that reported hindfoot pain (Fig 2), 4 noted pain localized to the talus [78,105,119,124]. In the midfoot, 10 of the 38 citations (Fig 2) reported pain localized to the medial midfoot [46,53,60,72,105,106,109,111,119,123] as well as pain localized to the navicular and two cuneiform bones [110]. In the forefoot area, four of the eight (Fig 2) included citations [9,84,87,128] identified pain specific to the metatarsal head region as well as tenderness at the first metatarsophalangeal joint (MTPJ) [128].

For pain related to joints outside of the foot, knee pain was the most reported symptom (n = 37) [4,9,16,21,39,47,49,53–55,57,60–62,65,66,68,69,72,74,75,77,82,87,88,91,97,99,100,103,109,111,128,130–133] (Fig 2) followed by, ankle (n = 24) [9,16,38,43,47,49,53,55,61,70,72,74,82,83,87,88,99,113,114,116,117,125,126,129] and hip (n = 13) [21,47,49,55,68,69,75,82,87,99,130–132]. While most of the citations reported these symptoms, no specific discussion or clarification on pain characteristics were given.

Pain in multiple body parts encompassed pain on activity (n = 48) [2,4,8,16,18,37,38,49,51,52,54,57,60,62,63,68,70,71,75,77,80,84,87,94,100,102,103,105–107,110–114,119,120,124–127,133–139] (Fig 2) and muscle pain (n = 2) [54,83]. Pain with walking (n = 19), playing sport (n = 9) [2,80,87,94,100,111,119,135,139] or general activity (n = 28) were most highly cited (Fig 2) followed by pain during running (n = 6) [37,51,68,75,103,114]. Pain was reported on activity and relieved by rest [107,112,113], which was made worse with activity [83] resulting in ceasing of activity [49] due to debilitating pain, limiting participation in sport, recreation and enjoyment of everyday activities [139].

Generalised pain (n = 33) [3,16,18,40,47,57,90,91,93,97,101,108,117,121,130,134,138–154] included reports of pain that did not describe or isolate body parts/structures or precipitating activities. Growing pains in the legs were less commonly identified (n = 4) [49,82,102,106] as a symptom.

Pain in the back was reported in 23 citations [4,21,39,43,49,54,55,57,66,68,69,75,79,82,87,88,90,99,111,130–133]. Of those, seven specifically identified the lower back as the site of symptoms [55,68,69,75,79,88,99]. No further characteristics were discussed.

Pain specific to sensory functions and pain was the least frequently reported category within the literature; including cramps (n = 8) [13,66,77,82,83,98,136,147], night pain (n = 5) [82,94,105,110,113] and inflammation (n = 4) [46,48,55,110]. Whilst characteristics of ‘cramps’ were not discussed, reassuringly, night pain was flagged as an uncommon and unusual symptom that requires further investigation [110,113]. Inflammatory pain of the tibialis posterior tendon was reported in one cohort study [147]. A cross-sectional study reported inflammation to the inner side of the foot [48] while a further two literature reviews reported inflammation (not specific to location) [46,110]. Inflammation was also considered an unusual symptom if present [110].

Radiating pain in a segment or region was reported specific to the posterior tibial tendon (n = 10) [21,37,49,55,66,76,85,88,90,147], however, no consistency in relating this to posterior tibial tendon dysfunction or insufficiency was observed. Similarly, pain was reported at the Achilles tendon in four studies (n = 4) [74,116,121,126] with no further discussion.

#### Reduced lower limb function.

Reduced function was reported in 102 (of 133) included citations. Reduced lower limb function was mapped across six categories: *sensation of muscle stiffness*; *gait pattern functions; functions of the joints and bones; stability of joints; spontaneous movement* and *muscle power functions.*

Sensations of muscle stiffness were the most reported function related symptoms (n = 67). Specifically, tightness of the Achilles tendon (n = 58) [1,3,4,13,16,45,47,53–55,59,61–63,66,69,71–73,76,81–86,88,89,91,95,98,100,105–108,110,112,113,115,119–121,123,125,126,128,130,133–136,138,140,143,147,150,154] (Fig 2), followed by ankle equinus (n = 5) [9,44,46,52,121], generalised muscle tightness (n = 3) [121,127,151] and hamstring tightness (n = 1) [45].

In relation to gait pattern functions, altered gait pattern (for example, reported as in-toeing or walking ‘funny’) was the most reported symptom (n = 35) [1,4,9,42,44,53,55,59–62,69,71,72,77–79,82,85,87,90,91,95,97,116,121,129,133,143,146–149,152,154] (Fig 2). Reduced speed was cited as a relative symptom of symptomatic flexible pes planus (n = 6) [3,9,21,42,44–46,155], with slower walking (n = 4) [9,21,42,155], running (n = 1) [42] and an overall reduced speed on an athletic field (n = 1) [45] reported. One case series [146] assessing children’s pain, function and shoe wear pre and post-surgical intervention reported those with symptomatic flexible flat foot will have difficulty walking on uneven surfaces, stairs, and ladders.

In relation to functions of the joints and bones, forty-one citations (n = 41) [1,3,4,17,19,21,38,47,48,51–53,57,60,69,71,78,79,82,84,90,92,96,98,106,108,109,121,129,132,134,138,141–144,149,154–157] reported an overall generalised reduction of lower limb function with no further characteristics discussed.

Symptoms identified regarding stability of joints included a reduction in overall balance and stability (n = 15) [3,16,18,46,47,60,61,71,74,78,92,116,121,134,135]. Four citations reported foot and ankle instability [18,61,74,135]. One randomized control trial reported medial foot instability [71], a literature review reported ankle instability [46] and one systematic review reported generalised instability [78]. Five quantitative studies reported poor overall balance [47,60,116,121,134].

Frequent injury was a symptom reported relative to spontaneous movement. Of the frequent injuries reported (n = 6) [16,38,54,70,74,135], ankle injuries, specifically, sprains were common (n = 4) [54,70,74,135] followed by overuse injuries (n = 2) [16,38].

In relation to muscle power function, muscle weakness (for example, reported as decreased muscle activation etc.) was noted in four (n = 4) [3,94,133,151] citations. Of those, two citations (one opinion piece and one literature review) reported overall muscle weakness [133,151] and one cross-sectional study reporting weakness specifically in the feet at night [94].

#### Fatigue and reduced endurance.

Fatigue and reduced endurance were reported in 60 (of 133) included citations. Fatigue and reduced endurance were reported and mapped across two categories: *fatiguability* and *general physical endurance.*

In relation to fatiguability, fifty-two citations (n = 52) [13,16,18,21,42,43,45,47,48,50,52,54,55,57,58,61,62,66,71,74,76,78,79,83,85–88,90,92,94,95,98,106,111,115–118,124,125,127,130,134–136,139,143,150,154,156] (Fig 2) identified fatigue as a common symptom in children with symptomatic flexible flat feet. Of those, 21 [13,16,21,43,47,57,62,66,76,78,79,83,98,117,125,127,134,136,139,150,154] reported generalised fatigue, while other citations focused specifically on locations of reported fatigue. Three individual citations noted fatigue specifically localized to only the feet and lower legs [55,88,95], whilst one literature review reported fatigue in the knee and back as well as foot and ankle [86].

Ten of the 52 citations identified activities that generated fatigue within this population [58,71,74,85,88,94,111,124,135,156]. The specific activities identified were walking (n = 4) [4,30,102,150], sports-based activities (n = 4) [4,19,30,120], playing (n = 1) [4] or prolonged standing (n = 1) [102].

In relation to general physical endurance, thirteen citations (n = 13) [1,4,9,21,61,62,69,72,77,83,97,130,140] reported on reduced endurance with activity. One literature review [9] highlighted those children in this population poorly performed physical activities poorly when compared to peers without flat feet. Another literature review [140] that discussed reduced endurance included a specific caution that any child with flat feet refusing to weight bear required health practitioner review.

#### Callus.

In relation to protective functions of the skin, callus formation, where skin on the plantar surface thickens in response to abnormal pressure or friction [158] was reported in 9 (of 133) [9,54,73,83,87,94,110,111,115] included citations. Only two citations [87,111], specified callus formation to the medial aspect of the foot while the rest did not specific the location of callus development.

### Body structure

#### Soft tissue changes.

Soft tissue changes were reported in 33 (of 133) included citations. Symptoms relating directly to soft tissue changes were mapped across two categories: *ligament and fascia of the lower limb* and *extra-articular ligaments, and, fasciae, extramuscular aponeuroses, retinaculae, septa, bursae, unspecified.*

In relation to ligament and fascia of the lower limb, thirty-three citations (n = 33) [1,4,8,36,45,46,55,60,67,68,70,71,74,81–84,89,94,96,100,106–108,110,113,115,126,128,133,135,139,148] (Fig 2) identified generalised ligament laxity (for example, reported as double jointed, very flexible etc.) as a symptom of symptomatic flexible pes planus. While 33 citations reported generalised ligament laxity, one Cohort study [115] reviewing clinical outcomes of children prior to calcaneal lengthening osteotomy procedure in Korea, identified one case of severe joint laxity.

### Friends

Friends was the second most common domain reportedly impacted for children with symptomatic flexible flat feet, with symptoms associated to participation related issues.

### Activities and Participation

#### Reduced participation.

Reduced participation was reported in thirty (n = 30) [3,9,21,42–44,48,49,52,54,57,59,60,63,65,77,82,87,94,98,100,101,111,121,123,133,136,146–148] (of 133) included citations.

Of the 133 included citations, four specifically identified a reduction in participation of daily activities [21,48,54,111], while three identified a reduction in running/jumping based activities [59,63,98]. One cross sectional study [94] identified children will present with a general lack of interest in sport or walking, while one opinion piece flagged that children will choose to not participate in activities entirely [82]. One literature review [100] highlighted if children with symptomatic flat feet did partake in sport, sport preferences were limited to only swimming or activities that required minimal impact.

### Major life areas

#### Reduced quality of life.

Reduced quality of life (QoL) was reported in twenty-four (n = 24) (of 133) included citations.

Although a less frequently mentioned, but nevertheless important symptom, reduced QoL was reported in 24 citations [3,16,18,19,37,42,48,50,59,70,78,84,89,92,120,130,133,136,137,140,142,145,148,157]. One cross sectional study [16] reviewing the quality of life in children with paediatric flexible flatfoot against children with ‘typically developing feet’, identified children with symptomatic flexible flat foot have a significantly impaired quality of life in comparison to children who have typically developing feet. Furthermore, another cross-sectional study [38] reported a reduction in quality of life being secondary to pain, functional impairments and overuse injuries caused by a flexible flat foot.

### Functioning

Functioning was the third most common domain reportedly impacted for children with symptomatic flexible flat feet, with symptoms associated to activity related issues.

### Moving around and walking and moving

#### Mobility related impairments.

Mobility impairments were reported in thirty (n = 30) (of 133) included citations. Symptoms relating directly to mobility related impairments were mapped across two categories: *moving around, other specified* and *walking and moving, other specified and unspecified.*

In relation to moving around, other specified, three main symptoms were identified, limping (n = 10) [58,74,77,90,97,103,115,129,133,140], increased tripping and/or falling (n = 5) [45,58,79,87,149] and increased clumsiness, (n = 2) [45,83]. Of the ten citations identifying limping as a symptom, one opinion piece [103] highlighted that limping occurred following strenuous activity.

Symptoms related to walking and moving, other specified and unspecified, were difficulty keeping up with peers (n = 7) [9,42,43,56,60,98,151] and difficulty playing sport (n = 12) [16,47,49,52,65,66,111,118,119,134,143,148]. One literature review [9] flagged children with symptomatic flexible flat feet poorly perform physical activities compared to their peers without flat feet.

### Family

Family was the fourth most common domain reportedly impacted for children with symptomatic flexible flat feet, with symptoms associated to environmental related issues.

### Products and technology for personal use in daily living

#### Shoe wear.

Shoe wear was reported in 25 (of 133) included citations.

While shoe wear is not regarded as a ‘symptom’ of symptomatic flexible pes planus, rather a sign, many citations (n = 25) [2,4,8,21,40,54,58,59,63,68,77,82,87,94,98,99,106,107,110,112,113,122–124,157] highlighted this as a finding. Most citations discussing shoe wear identified uneven shoe wear as most common (n = 16), followed by rapid shoe wear (n = 5) and discomfort or difficulty with shoe fitting (n = 4). While one citation specifically noted increased breakdown to the sole at the medial plantar forefoot region [97], another citation reported increased shoe wear at the posterior medial heel area [86].

### Future

Future was the least common domain reportedly impacted for children with symptomatic flexible flat feet.

In relation to future, two citations (n = 2) [18,135] reported that early muscle fatigue and foot and ankle instability (for example, reported as ankle sprains, tripping over etc.) can lead to capsule-ligamentous strain/sprain and imbalance which may be a precursor for progressive deformity and degenerative arthropathy during adulthood.

## Discussion

To date no known review has systematically mapped symptoms associated with symptomatic flexible flat feet. This review has identified that the symptoms of flat feet were very broad. Our findings were mapped against the F-words and the ICF-11 was incorporated allowing the utilization of a well-established framework to describe and organize the information relative to functioning and disability. Organizing information in this way gives structure and order to the plethora of signs and symptoms we found relating to symptomatic flat feet. A key finding from this review indicates that symptoms of symptomatic flexible flat feet can broadly be categorized into five of the six F-word categories: *Fitness, functioning, friends, family* and *future.* The impact on *fun* as the sixth category was not captured in this review. Other relevant findings highlight that pain, was overwhelmingly, the most reported symptom, albeit at varying locations, structures, and during differing activities. Other frequently reported symptoms included generalised fatigue, reduced participation and endurance and Achilles tendon tightness. In addition to commonly reported symptoms, several uncommon symptoms associated with symptomatic flexible flat foot were also reported, such as night pain or cramps. This review also highlights the lack of consensus and clarity of reported and known symptoms relative to this foot posture.

The included number of citations in this scoping review (>100) provide insights into the popularity of symptomatic flexible flat foot. This number is in stark contrast to the lack of clarity and consensus on the categorization of types of flat feet and the reporting of symptoms. Only one citation [16] clearly defined the symptomatic flexible flat foot and outlined varied means of subjective and objective measurement (interviews, clinical measure assessments and functional tests). The reasons so many symptoms are associated with symptomatic flexible flat foot could be due to several reasons. First, perhaps symptomatic flexible flat foot truly does present with many and varied symptoms, which may occur independently or collectively, based on the individual. Furthermore, despite clear evidence that a child’s foot posture is expected to appear visually ‘flat’ when compared to adults, ambiguity exists on what should or shouldn’t be classified ‘flat feet’. Therefore, clinicians and parents alike may be combining any and all reported or noted symptoms reported by children in relation to their lower limb as directly related to their ‘flat foot’ presentation, even when that child’s foot posture is within the expected range for flatness. This may explain, to some extent, why most citations did not include a diagnosis or assessment method that could validate their classification of flat feet. Currently, the symptoms of symptomatic flat feet lack clear boundaries which creates confusion for clinicians. This could be addressed, however, it would require large scale, longitudinal cohort studies.

While a broad array of symptoms was reported in the literature, it could be allocated into five of the six categories within the ‘F-words’ (*fitness, functioning, friends, family,* and *future)*. One citation [21] from this review identified 19 different symptoms which could be categorized into four of the ICF-11 categories (*fitness, functioning, friends* and *family). Fitness* and *friends* were overwhelmingly the most used categories across this review. More specifically, pain was the most reported symptom. Within the literature, pain is often cited as a common symptom of many paediatric conditions, i.e., growing pains, muscular strain, tendinopathies [70,159,160] and more lower limb specific conditions such as apophyseal osteochondrosis [161], tarsal coalitions and accessory bones of the feet [70]. Outside of these discreet conditions, there may be other explanations for why pain was repeatedly reported by children. First, rather than using various terms, children may be more likely to unify all their symptoms as pain, as this may be something they are familiar with [162]. The second reason is the unidimensional view of how clinicians/researchers may see this from a biomedical perspective. Whilst there is evidence that children around the age of 8 years [163] can report pain accurately; however, in our review it was more commonly reported throughout opinion pieces, which may potentially, reflect clinician expectations of presentations of flat feet. Finally, pain may be the initial presenting symptom in children with symptomatic flexible flat foot, which over time, may develop into other symptoms (e.g., fatigue, difficulty keeping up with peers etc.).

Outside of pain, it is important to note that other musculoskeletal conditions were also reported within the findings of this review [159,160]. Symptoms reportedly associated with a flat foot posture include fatigue, reduced lower limb function, reduced strength and endurance and activity limitation, potentially having a negative impact on children’s overall quality of life [19]. These findings are consistent with previous literature [1,4,20,108,129]. Less commonly reported symptoms included callus formation, uneven shoe wear and cramps. Whilst there is an unknown correlation between paediatric and adult flat foot, we do know that adults report callus 3.5 times more if they have flat feet [164]. The breadth of non-pain-based symptoms lends support to the context that a diagnosis of symptomatic flexible flat foot can be made in the absence of pain.

### Limitations

Firstly, the literature searches were limited to the English language only. While this may have resulted in language bias, the use of comprehensive search strategy meant the review identified multiple studies from countries where English is not the first language. Secondly, symptoms that could be categorized into *fun* (personal factors) were not captured within the literature reviewed. This may have been due to the subjective nature of categorization and interpretation of symptoms by the review team or due to a reduced focus of ‘fun’ related symptoms relative to symptomatic flexible flat feet. We used strategies to ensure rigour and robustness of this process and categorization of symptoms was mapped based on Rosenbaum and Gorter [30] to mitigate this challenge. However, the allocation of symptoms remained subjective to the authorship team. Several citations were excluded due to a lack of definition of symptomatic flexible flat foot. When extracting relevant data, the discrepancy in definition and classification lead to a reduced number of overall included citations. To reduce the risk of this occurring, the authorship team, along with a librarian, aimed to include as many truncations, key words, and free text forms as possible. Finally, although there were 133 citations included in this review, only a handful of these citations were research which had a specific focus on measurement of symptoms of paediatric flexible flat feet. Majority of the literature merely highlighted these symptoms, without adequate or formal measurement.

### Recommendations

Based on the findings from our scoping review, we propose three key recommendations for clinical practice. First, given the symptoms associated with symptomatic flexible flat foot are numerous, diverse, and variably reported, we recommend clinicians recognise this complexity when treating children with symptomatic flexible flat feet. Second, while pain was the most reported symptom, as it is unclear if symptoms present in isolation, co-exist simultaneously or if one symptom progresses to another (e.g., pain leading to reduced function), a thorough and detailed clinical examination is required. Finally, as there is lack of clarity on the reporting of the categorization of types of flat feet and associated symptoms, future research is required to address these knowledge gaps. These can include standardised frameworks for categorization of types of flat feet and the reporting of symptoms, population-based cohort studies which follow up children over many years to identify when symptoms appear, the progression of the symptoms, and the actors that underpin the development of symptoms.

## Conclusion

This scoping review underscores the complexity and variability of symptoms related to paediatric symptomatic flexible flat feet. By mapping the 42 identified symptoms using the ‘F-words’ framework, we have identified that symptoms impact various aspects of a child’s life, including *fitness, functioning, friends, family and future*. Notably, pain presented as the most prevalent symptoms, while other symptoms such as altered gait pattern and reduced quality of life further highlight the multifaceted nature of flexible flat feet. Future research can focus on clarifying the categorization of flat feet and associated symptoms through longitudinal studies that track symptom progression.

## Supporting information

S1 Appendix Appendix 1. Medline Search. Appendix 2. Data Extraction. Appendix 3. Emcare Search. Appendix 4. Embase Search. Appendix 5. The Cochrane Library Search. Appendix 6. UpToDate Search. Appendix 7. Scopus Search. Appendix 8. SportsDiscus Search. Appendix 9: Overview of data mapped against the ICF domains and sub-domains of reported symptoms
(DOCX)
