# Supplementary material for: The F-words relating to symptomatic flexible flat feet: A scoping review
Source: PLoS One. 2025 May 7;20(5):e0320310. doi: 10.1371/journal.pone.0320310 (PMC12057978; doi:10.1371/journal.pone.0320310)
Supplement: S1 Appendix — Appendix 1. Medline Search. Appendix 2. Data Extraction. Appendix 3. Emcare Search. Appendix 4. Embase Search. Appendix 5. The Cochrane Library Search. Appendix 6. UpToDate Search. Appendix 7. Scopus Search. Appendix 8. SportsDiscus Search. Appendix 9: Overview of data mapped against the ICF domains and sub-domains of reported symptoms (DOCX) [file pone.0320310.s001.docx]

Appendices

| **Database** | **Searches** |
| --- | --- |
| Medline (Appendix 1) | 1. Symptom Assessment/ 2. (symptom* r indicator*).ti,ab,kf. 3. (fatigue or pain* or quality of life or trip* or clumsiness* or clumsy or endurance or hypermobile or hypermobility).ti,ab,kf. 4. 1 or 2 or 3 5. Flatfoot/ 6. Flexible flatfoot.mp. 7. (flat foot or flatfoot or flat feet or flatfeet or flexible flatfoot or flexible flatfeet or flexible flat foot or flexible flat feet or pes planus or pes planovalgus).ti,ab,kf. 8. 5 or 6 or 7 9. Child/ 10. Adolescent/ 11. (child* or newborn* or infant* or neonate* or baby or babies or toddler* or preschool child* or adolescent* or teen* or juvenile* or youth* or minor or paediatric* or pediatric*).ti,ab,kf. 12. 9 or 10 or 11 13. 4 and 8 and 12 14. Limit 13 to (English language and humans) |
| Emcare (Appendix 3) | 1. Symptom Assessment/ 2. (symptom* r indicator*).ti,ab,kf. 3. (fatigue or pain* or quality of life or trip* or clumsiness* or clumsy or endurance or hypermobile or hypermobility).ti,ab,kf. 4. 1 or 2 or 3 5. Flatfoot/ 6. Flexible flatfoot.mp. 7. (flat foot or flatfoot or flat feet or flatfeet or flexible flatfoot or flexible flatfeet or flexible flat foot or flexible flat feet or pes planus or pes planovalgus).ti,ab,kf. 8. 5 or 6 or 7 9. Child/ 10. Adolescent/ 11. (child* or newborn* or infant* or neonate* or baby or babies or toddler* or preschool child* or adolescent* or teen* or juvenile* or youth* or minor or paediatric* or pediatric*).ti,ab,kf. 12. 9 or 10 or 11 13. 4 and 8 and 12   Limit 13 to (English language and humans) |
| Embase (Appendix 4) | 1. Symptom Assessment/ 2. (symptom* r indicator*).ti,ab,kf. 3. (fatigue or pain* or quality of life or trip* or clumsiness* or clumsy or endurance or hypermobile or hypermobility).ti,ab,kf. 4. 1 or 2 or 3 5. Flatfoot/ 6. Flexible flatfoot.mp. 7. (flat foot or flatfoot or flat feet or flatfeet or flexible flatfoot or flexible flatfeet or flexible flat foot or flexible flat feet or pes planus or pes planovalgus).ti,ab,kf. 8. 5 or 6 or 7 9. Child/ 10. Adolescent/ 11. (child* or newborn* or infant* or neonate* or baby or babies or toddler* or preschool child* or adolescent* or teen* or juvenile* or youth* or minor or paediatric* or pediatric*).ti,ab,kf. 12. 9 or 10 or 11 13. 4 and 8 and 12   Limit 13 to (English language and humans) |
| The Cochrane Library (Appendix 5) | 1. MeSH descriptor: [Symptom Assessment] explode all trees 2. (symptom* OR indicator* OR fatigue* OR pain* OR quality of life OR trip* OR clumsiness* OR clumsy OR endurance OR hypermobile OR hypermobility):ti,ab,kw 3. #1 or #2 4. MeSH descriptor: [Flatfoot] explode all trees 5. Flat foot OR flatfoot OR flat feet OR flatfeet OT flexible flatfoot OR flexible flatfeet OR flexible flat foot OR flexible flat feet OR pes planus OR pes planovalgus 6. #4 or #5 7. MeSH descriptor: [Child] explode all trees 8. MeSH descriptor: [Adolescent] explode all trees 9. (child* OR newborn* OR infant* OR neonate* OR baby OR babies OR toddler* OR preschool child* OR adolescent* OR teen* OR juvenile* OR youth* OR minor OR paediatric* OR pediatric*):ti,ab,kw 10. #7 or #8 or #9 11. #3 and #6 and #10 |
| UpToDate (Appendix 6) | Paediatric flatfoot |
| Scopus (Appendix 7) | Symptom* OR symptom AND assessment* OR indicator* OR sign*  AND  Flatfoot OR flatfeet OR flat AND foot OR “pes planus” OR “flexible flatfoot”  AND  Child* OR infant* OR baby OR babies OR toddler* OR “preschool child” OR juvenile* OR youth* OR minor OR paediatric* OR pediatric |
| SportsDiscus (Appendix 8) | ( “symptom” OR “indicator” OR “fatigue” OR “pain” OR “quality of life” OR “trip” OT “tripping” OR “clumsiness” OR “clumsy” OR “endurance” OR “hypermobile” OR “hypermobility” )  AND  ( “flatfoot” OR “flat foot” OR “flatfeet” OR “flat feet” OR “flexible flatfoot” OR “flexible flatfeet” OR “flexible flat foot” OR “flexible flat feet” OR “pes planus” OR “pes planovalgus” )  AND  ( “child” OR “newborn*” OR “infant*” OR “neonate*” OR “baby” OR “babies” OR “toddler*” OR “preschool child*” OR “juvenile*” OR “adolescent*” OR “youth*” OR “paediatric*” OR “pediatric*” ) |
